# Translation of tobacco policy into practice in disadvantaged and marginalized subpopulations: a study of challenges and opportunities in remote Australian Indigenous communities

**DOI:** 10.1186/1478-4505-10-23

**Published:** 2012-07-28

**Authors:** Jan A Robertson, Katherine M Conigrave, Rowena Ivers, Kim Usher, Alan R Clough

**Affiliations:** 1 School of Public Health, Tropical Medicine and Rehabilitation Sciences, James Cook University, PO Box 6811, Cairns, Queensland, 4870, Australia; 2 School of Nursing, Midwifery & Nutrition, James Cook University, PO Box 6811, Cairns, Queensland, 4870, Australia; 3 University of Sydney Central Clinical School, Royal Prince Alfred Hospital, Missenden Road, Camperdown, NSW, 2050, Australia; 4 Addiction Medicine & School of Public Health, Sydney Medical School, The University of Sydney, Sydney, New South Wales, 2006, Australia; 5 Graduate School of Medicine, University of Wollongong, Wollongong, New South Wales, 2522, Australia

## Abstract

**Background:**

In Australia generally, smoking prevalence more than halved after 1980 and recently commenced to decline among Australia's disadvantaged Indigenous peoples. However, in some remote Indigenous Australian communities in the Northern Territory (NT), extremely high rates of up to 83% have not changed over the past 25 years. The World Health Organisation has called for public health and political leadership to address a global tobacco epidemic. For Indigenous Australians, unprecedented policies aim to overcome disadvantage and close the 'health gap' with reducing tobacco use the top priority. This study identifies challenges and opportunities to implementing these important new tobacco initiatives in remote Indigenous communities. Methods: With little empirical evidence available, we interviewed 82 key stakeholders across the NT representing operational- and management-level service providers, local Indigenous and non-Indigenous participants to identify challenges and opportunities for translating new policies into successful tobacco interventions. Data were analysed using qualitative approaches to identify emergent themes.

**Results:**

The 20 emergent themes were classified using counts of occasions each theme occurred in the transcribed data as challenge or opportunity. The 'smoke-free policies' theme occurred most frequently as opportunity but infrequently as challenge while 'health workforce capacity' occurred most frequently as challenge but less frequently as opportunity, suggesting that policy implementation is constrained by lack of a skilled workforce. 'Smoking cessation support' occurred frequently as opportunity but also frequently as challenge suggesting that support for individuals requires additional input and attention.

**Conclusions:**

These results from interviews with local and operational-level participants indicate that current tobacco policies in Australia targeting Indigenous smoking are sound and comprehensive. However, for remote Indigenous Australian communities, local and operational-level participants' views point to an 'implementation gap'. Their views should be heard because they are in a position to provide practical recommendations for effective policy implementation faithful to its design, thereby translating sound policy into meaningful action. Some recommendations may also find a place in culturally diverse low- and middle-income countries. Key words: tobacco policy implementation, challenges, opportunities, remote Indigenous Australian communities**.**

## Introduction

In Australia generally, tobacco use continues to decline with only 15.1% of the population aged over 14 years smoking daily [1]. However, there are impoverished subpopulations in Australia, disadvantaged in similar ways to those living in low income countries, where this decline is not occurring. For example, daily smoking prevalence among Australia’s half a million Indigenous people is estimated to be 47.7% in those aged 18 years and over, with higher prevalence in remote communities (53%) than in major cities (42%). For Indigenous Australians, there is evidence for a small but statistically significant decline in smoking rates after 2002 from 53% to 50%. However, in remote Indigenous communities in the ‘Top End’ of the Northern Territory (NT), where around 26,000 Indigenous people live, extraordinarily high smoking rates of 65%–83% have changed little over the last quarter of a century [2-4]. Recent data available from surveys conducted in three communities in this region in 2008/09 showed that 76% ( from 71%-82%) of participants (aged ≥16 years) were self-reported current smokers [5]. Despite these extremely high levels, an encouraging finding was that 58% of the current smokers were thinking of quitting and 17% were attempting to quit at the time of the survey or had tried to quit in the recent past [6]. A widespread desire to quit suggests substantial opportunities in these communities to reduce tobacco use with effective programs if they can be implemented.

In 2008, the World Health Organisation (WHO) outlined a package of tobacco control policies to address the global epidemic of tobacco use calling for political and public health leaders to make the implementation of these policies a matter of highest priority [7]. These policies focus on monitoring of tobacco use, and on prevention; providing both warnings regarding tobacco-related harms and protection from second-hand smoke; offering cessation support; enforcing advertising and sponsorship bans and raising tobacco taxes. Many components of the current Australian policy environment surrounding efforts to reduce tobacco use by Indigenous Australians align well with WHO policies and are unprecedented in Australia. In 2008, the Council of Australian Governments signed off on the National Indigenous Reform Agreement, committing all Australian States and Territories to ‘Closing the Gap’ in Indigenous disadvantage [8]. Associated initiatives are backed by significant resource allocation for the five-year period 2009 to 2013, through National Partnership Agreements (NPA) between Commonwealth, State and Territory governments [9]. The NPA on ‘Closing the Gap’ in Indigenous Health Outcomes specifically identifies “Tackling Smoking” as the first of five priority initiatives to address chronic disease risks [9]. The widespread desire to quit among smokers documented in our recent surveys in remote communities suggests a high need to successfully implement sound programs in these localities arising from the new tobacco policies. The current policy environment in Australia provides the first major opportunity to reduce extraordinarily high smoking rates in remote Indigenous communities. Effective implementation will be reliant on successful translation of apparently sound policies into sustainable programs with practical actions.

In a recent review of evaluated international alcohol, tobacco and other drugs interventions at the community level, Geisbrecht and Haydon [10] recommended that government and funding bodies acquire local knowledge to inform policy relating to community interventions. In addition, a ‘Better Practice Guide’ for government policy and program implementation advises that policy development should be informed of “*risks, challenges and practical aspects that may have an impact on implementation*” [11]. Guided by these recommendations, to identify any ‘implementation gaps’, we focused on *challenges* and *opportunities* for program implementation in interviews with stakeholders mainly working at the operational level in remote communities in Arnhem Land, in the NT’s ‘Top End’.

## Methods

### Setting

**Figure 1:**
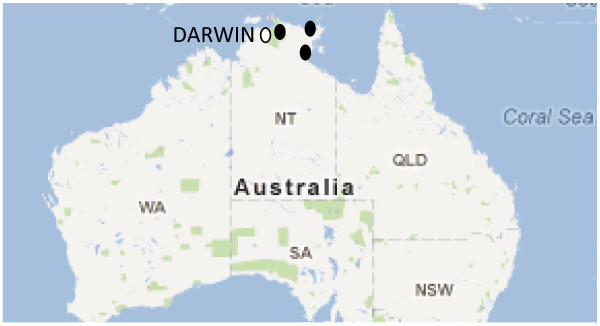
**Three study communities.** Top End Tobacco Project, Arnhem Land, Northern Territory.

The research reported here is part of the ‘Top End Tobacco Project’ (TETP), a five year multiple-component, community-action intervention study to reduce tobacco smoking in three remote Aboriginal communities in Arnhem Land, a region east of Darwin in the NT’s ‘Top End’ (Figure 1). The communities have populations ranging from around 800 to 2100. Tobacco is locally available from a small number (n = 10) of community-based retail outlets. They are discrete and isolated communities, cut off from each other and the main regional centres in the NT’s ‘Top End’ by sea or by wet season river rises. Socially, culturally and linguistically distinct, local languages and many traditional cultural practices are largely intact.

Remote communities in the region are highly dependent on regional centres for the management and delivery of services. Service providers who work at the community level travel from these centres, covering large distances by road or air with the frequency and duration of their community visits often constrained by flooded roads in the wet season, lack of accommodation and limited travel budgets. The NT’s capital city of Darwin and the smaller regional centres of Nhulunbuy and Katherine with populations between 4000 and 7000, respectively, are main service centres for the study communities (see Figure 1). Two of the three study communities have health clinics operated by Aboriginal community-controlled health services based in the regional centres and the third has a clinic run by the NT Government’s health department.

### Policy environment

Contributing to the ‘Close the Gap’ targets is the 2009 National Partnership Agreement on Remote Service Delivery (NPARSD) in remote communities [12]. The agreement incorporates coordination between Commonwealth and Territory governments and encourages active participation by Indigenous community members including their involvement in the development of Local Implementation Plans (LIPs). Of particular relevance to this paper, currently 10 of the 15 NT communities involved in this initiative include in their LIPs addressing high prevalence of tobacco use as a priority health action [personal communications JR 2012: M Klopper, Acting Area Manager, Regional Operations Centre, NPARSD]. NT Government reforms in July 2008 included the amalgamation of fifty-three remote community councils into eight larger shires [13]. This shifted decision-making and management about most local community-level matters to the regional centres with implications for service delivery generally, and for implementing tobacco policies and programs at the community level in particular. Significantly, a smoke-free policy for all its facilities, including those in remote communities, was launched by the NT Department of Health and Families in 2009 [14].

### Participants and sampling

Purposive sampling was used to recruit stakeholders with either a mandate or an interest in reducing tobacco use in the NT’s Indigenous population. In the three study communities of the TETP, locally-based service providers in local government, education, health and allied services, community and church leaders and other community members were approached for interview. Incorporating a ‘relational’ perspective for qualitative research as described by Cummins et al. [15], we extended the geographical reach of the interviews beyond the study communities. We sought the views of those based in regional centres external to the study communities who were working in a similar context. We also interviewed others across the jurisdiction who, although geographically distant, were in relationships of power or influence for addressing tobacco use in these settings*.* Included were elected members of the NT Government and relevant policy advisors.

In order to achieve saturation, we coupled the purposive sampling with a snowball approach asking each participant at the end of the interview to recommend further participants. To understand opportunities and challenges for policy implementation at the community level in particular, we ensured that the sample included the main frontline service providers, defined as those who worked at the public interface in the community. Frontline service providers also included those based in a regional centre but who visited the communities on a regular basis in order to provide services such as nutrition education, substance misuse and health promotion. A target of 50 interviews was initially set aiming for a balance between Indigenous and non-Indigenous participants. Apart from two phone interviews, all interviews were conducted face-to-face in participants’ workplaces for their convenience or where participants felt most comfortable. The interviews were mainly one-on-one, however some discussions were held in small groups when the participants preferred this. Indigenous participants tended to prefer group discussion. The interviews were conducted in plain English (by JC and AC) with limited use of local languages for key words and phrases. Interviews lasted from 20 minutes up to 90 minutes, in as much depth as participants were prepared to offer.

### Interview framework

An unpublished pilot study conducted in one locality in the region in 2006–2007 provided preliminary indications of possible key themes that may emerge in further research. These included: ‘competing priorities for service providers’, ‘current policy environment’, ‘cultural issues’, ‘accessible and appropriate health promotion resources’ and ‘treatment services’. This pilot project suggested that these could be broadly grouped as ‘*challenges’* and/or ‘ *opportunities’*. On this basis, an ‘ *opportunity’* was defined for the purposes of this study as a chance to enhance tobacco intervention components and programs. A ‘ *challenge’* was defined as a potential or actual limitation to implementing tobacco interventions. Using a semi-structured interview approach, participants were invited to discuss ‘ *opportunities*’ and ‘ *challenges*’ to addressing tobacco-related issues. Participant responses were transcribed from hand-written notes taken during the interviews.

### Data analysis

We analysed data using a combination of qualitative and simple quantitative approaches. Following standard qualitative data analysis procedures, the data analysis program Nvivo [16,17] assisted to extract responses relating to the domains of ‘*challenges*’ and ‘ *opportunities*’ from interview transcripts. Emergent themes were derived using a constant comparative method, i.e. moving repeatedly between themes and the transcribed text [18]. The themes were verified by inter-coder agreement following independent analyses (JR and AC). To assist with cross-case comparisons, each interview participant was classified as either:

frontline service provider, Indigenous (FLSPI);

frontline service provider, non-Indigenous (FLSPNI);

non-frontline service provider, Indigenous NFLSPI) or

non-frontline service provider, non- Indigenous (NFLSPNI).

Other attributes allocated included: ‘location of work base’, whether a ‘resident in the community’, ‘experience in their field’, ‘key function’ and ‘government affiliation’.

To summarise the ‘*opportunities’* and ‘ *challenges’* in the data as a whole, a scatter-plot was prepared comparing the number of occasions an ‘ *opportunity’* was also mentioned as a ‘ *challenge*’. This permitted a graphical assessment of the relative strength of views among those interviewed and how each ‘ *challenge’* and *‘opportunity’* clustered with others.

### Ethics approvals

Ethics approval for the study was provided by the Human Research Ethics Committee of James Cook University and the NT Department of Health and Families and Menzies School of Health Research. Permissions to visit communities were obtained from local Aboriginal community councils and from the Northern Land Council, as required under the NT Aboriginal Land Act (1980). Australia’s National Health and Medical Research Council protocols for research with Indigenous Australians were followed. All participation was voluntary and all of those interviewed signed consent forms.

## Results

A total of 82 key stakeholders participated in 65 interview sessions conducted over 13 months commencing March 2009. JR conducted 54 interview sessions and AC conducted 11. Of these, 17 interview sessions were completed with groups of from two to seven participants. Interviews ceased when saturation of information was reached, indicated when new participants began to consistently refer us to the same stakeholder individuals or groups and when similar information was provided with the topics raised by participants converging.

### Participant attributes

A majority (62%, n = 51) of the 82 participants were non-Indigenous. Of the total group of 82 participants, 62 (76%) were frontline service providers and of these, around half (n = 30) were Indigenous. Of the 20 (24%) non-frontline service providers interviewed, only one identified as Indigenous. Of the total group of 82, 63 (76%) had greater than five years experience working in remote communities or were themselves community residents.

At the time of interviews, prevention and/or treatment of tobacco-related harms was, at the community level, just one component of the core business of health service providers. Of all those interviewed, 49 (60%) worked in health. Of the 62 frontline service providers interviewed, 24 (39%) were visitors, i.e. they were not resident in the communities. Service providers resident in the communities included health staff, teachers, shire employees and church leaders. Non-frontline service providers, not community-based, were interviewed in regional centres and these included: executive level managers, program managers, researchers, those with a specific advocacy role and politicians at the regional and NT Government levels including an NT Government Minister. With regard to government affiliation, 33 (40%) of those interviewed were employed in the non-government sector. The majority of these were health employees working in community-controlled health services.

### Themes emerging from interviews

Twenty major themes emerged as both *opportunities* and *challenges.* It was first thought that there would be strong differences between the views of the Indigenous and non-Indigenous participants about perceived *challenges* and *opportunities*. Although numbers were too small for meaningful statistical significance testing or quantitative assessment of levels of agreement for each theme, we cross-tabulated the frequencies with which frontline service providers and Indigenous people mentioned each theme as an *opportunity* and/or a *challenge*. In these cross-tabulations it was more often the case that the counts for Indigenous and non-Indigenous participants were similar for each theme, but there were consistently greater differences between the front-line and non-frontline service providers, as Table 1 illustrates. This broad agreement of views regarding an *opportunity* or a *challenge* among front-line service providers was evident regardless of whether participants were community residents or visitors, Indigenous or non-Indigenous. Furthermore, front-line service providers provided the more considered and detailed responses to interview questions including practical suggestions for policy implementation.

**Table 1:** Occasions of mentions of themes: Group 1

**THEME**	**PARTICIPANT STATUS**	***OPPORTUNITY***		***CHALLENGE***	
		**Frontline**	**Non-frontline**	**Frontline**	**Non-frontline**
Smoke-free policies	Indigenous	38%	6%	25%	0%
	Non-Indigenous	34%	22%	75%	0%
Smoking cessation support	Indigenous	44%	0%	26%	11%
	Non-Indigenous	48%	8%	58%	5%
Health promotion activities	Indigenous	25%	5%	31%	0%
	Non-Indigenous	50%	20%	46%	23%
Target groups	Indigenous	58%	0%	34%	33%
	Non-Indigenous	37%	5%	33%	0%
Policy environment	Indigenous	25%	19%	50%	12%
	Non-Indigenous	25%	31%	25%	13%
Health workforce capacity	Indigenous	13%	0%	8%	5%
	Non-Indigenous	80%	7%	54%	33%

Proportion of mentions as either an *opportunity* or a *challenge*, comparing Indigenous and non-Indigenous participants with frontline and non-frontline service providers.

**Figure 2:**
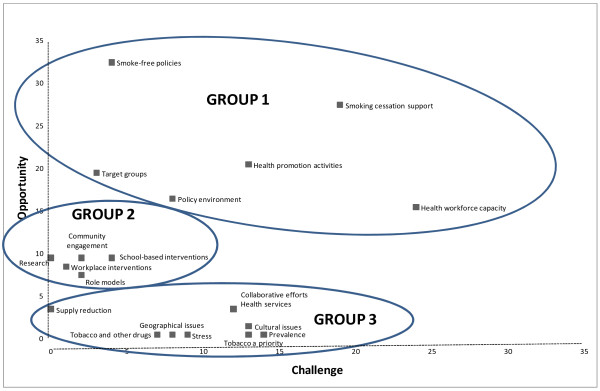
**Occasions themes (n = 20) were mentioned as an opportunity (y- axis) or as a challenge (x-axis).** The scatter plot of occasions of mention of themes as an opportunity and as a challenge suggests three groups. Group 1: frequently seen by participants as an opportunity but along a continuum of challenge; Group 2: often seen as an opportunity but not often as a challenge; Group 3: not often seen as an opportunity but often seen as a challenge.

Figure 2 is a scatter plot of the frequencies with which each of the themes was mentioned in the transcribed data as an *opportunity* and as a *challenge* allowing us to group the themes. Inspection of Figure 2 suggests three groups each discussed in turn:

Group 1: Frequently seen by participants as an *opportunity* but along a continuum of challenge.

Group 2: Often seen as an *opportunity* but not often as a *challenge*.

Group 3: Not often seen as an *opportunity* but often seen as a *challenge*.

#### Group one: Frequently seen as opportunity along a continuum of challenge

Themes in this group are described in descending order of *opportunity* namely: ‘smoke-free policies’, ‘smoking cessation support’, ‘health promotion activities’, ‘target groups’, ‘policy environment’ and ‘health workforce capacity’ (Figure 2).

**‘Smoke-free policies’** was most frequently mentioned by all participants as an *opportunity* and rarely as a *challenge*. Among the sub-themes linked with this *opportunity*, three types of spaces were identified where the policies are or need to be applied, namely workplaces (especially health clinics), peoples’ homes and public spaces. Favourably mentioned as an important initiative was the NT Department of Health’s introduction of a smoke-free workplace policy in July 2009 [14]. But there was a broader view expressed regarding the scope of smoke free policies. The notion that “*all homes should be smoke-free“* (FLSPI) was expressed at least once in each community by community members. These comments usually reflected a concern for reducing the risk of passive smoking for babies, young children and elderly people suffering from chronic diseases. Practical suggestions for smoke-free public spaces included commonly-frequented areas such as the front of community stores or sports and recreation areas. Informal gambling circles where participants noted that individual tobacco consumption rises markedly with the “stress” of card games were also recommended to be “ *declared smoke-free*” (FLSPI).

Seen as a *challenge*, lack of enforcement of existing policies at the community level was mentioned mainly by non-Indigenous, frontline service providers who called for more support from agencies external to the community to address a contentious and potentially divisive issue: *“…they don’t acknowledge that it’s difficult for people who live and work in the community to approach local services and organizations like the store and advocate for change*” (FLSPNI) and “.. *it’s impossible to enforce the smoke-free policy around council buildings – no-one takes notice. It’s also difficult to enforce no smoking in vehicles”* (FLSPI).

**‘Smoking cessation support’** was identified more frequently as an *opportunity* particularly by FLSP’s, but was also often regarded as a *challenge*. Very little cessation support for individuals was available in communities and usually accessible only through local health clinics. Recommendations included: “ *a QUIT* (telephone counseling) *NT specific service that accommodates, drives and supports interventions from a locally based area”* (FLSPNI) and “ *access to frequent follow-up especially in the first month*” (FLSPNI). With mainly nicotine patches available in communities, there were calls for a wider range of NRT products (e.g. nicotine gums) in order to provide: “ *opportunity for combination therapy that may be more effective for highly dependent smokers*” (FLSPNI). Support models such as telephone and website quit support services in their current form were thought to be “… *culturally inappropriate and logistically impossible*” (FLSPNI) and that “ *the government delivers the message* (quit smoking) *but doesn’t provide the support needed to make the change*” (FLSPI).

Homelands or outstations are small isolated settlements located on traditional lands outside of the larger communities where there is a return to traditional activities like hunting and fishing. Often access to tobacco is constrained. Local community members noted these environments provided an *opportunity* to quit: “ *Some go to the outstations to try to forget about ngarali* [tobacco]”(FLSPI). A local government shire manager stated there was also an “ *opportunity in the workplace to offer support and quit groups*” (FLSPNI).

**‘Health promotion activities’** in the data refer to local efforts to increase health literacy and strengthen community action relating to tobacco. This was often mentioned as an opportunity and less often mentioned as a *challenge* (Figure 2)**.** It was recommended that health promotion activities should be delivered by community people in conceptually and culturally appropriate ways. “ *Generally, approaches for education don’t take into account cultural attitudes. Smoking is embedded, normalized. There are cultural responsibilities, fatalism*” (FLSPNI). Community members in particular felt that more information regarding the poisons found in cigarettes and about smoking-related harms would help to inform healthier lifestyle choices: “ *Tobacco is mulkuru* [strange] *. It’s a manymak* [good] *thing. But people also know it’s not good for them. But they don’t know why. Provide the real story about the damage”* (FLSPI).

The almost universal practice of mixing cannabis with tobacco among cannabis users, already documented in the study region [19], was referred to several times and recommendations included that this practice should be acknowledged and addressed in both education and quit support. Further recommendations for local health promotion activities included: using expired breath carbon monoxide monitors (as used by the research team at time of community tobacco surveys); providing information on addiction including withdrawal; resources in local language and *“talking in a good way with smokers, not being angry or judging them*” (FLSPI).

**‘Target groups’**: Pregnant young women were the most frequently mentioned target group for tobacco interventions by both community members and service providers at the frontline. “A s *erious issue is the young pregnant women smoking – everyone notices that and that it’s not a good thing to do but they all just let it slide”* (FLSPNI). The best opportunity for engaging with this group was thought to be through antenatal clinics, however there was uncertainty as to whether these opportunities were being used to their full potential.

Targeting families was frequently strongly advised across the range of interviewees, recommending strengths-based messages: *“There are ways of spending instead, support your family. Look at tobacco from a family perspective – second hand smoke, health of kids”* (FLSPNI).

Young age of tobacco uptake was of great concern generally: ”*Prevention, prevention, prevention – work on the new generation”* (FLSPI). Interventions targeting pre-school children and school students were recommended with the recognition that ” e *ducation at schools needs to coincide with whole of community campaigns asinfluences at home will far outweigh the influence of any changes at the school or elsewhere*” (FLSPNI). Although schools were seen by Indigenous community members as the prime location for prevention activities, most educators interviewed expressed reluctance to take on the issue of tobacco, arguing that the curriculum was already overloaded by demands for extra programs such as “ *breakfast and feeding programs and complex reporting requirements*” (FLSPNI).

**‘Policy environment’:** The contemporary health policy environment at the national and NT level was frequently mentioned as an *opportunity* (n = 16) to address the high rates of tobacco use in these localities (Figure 2). “*The new COAG* [Council of Australian Governments] *announcement around tobacco allocation dollars….previous funding has been generic drug and alcohol funding. This is specifically tobacco”* (NFLSPI). *“Close the Gap efforts* [on indigenous disadvantage] *– especially for generations to come”* (FLSPI). Commonwealth subsidies on NRT patches were also mentioned as an *opportunity.* Jurisdiction-level policies mentioned as opportunities included local government reforms and the introduction of a jurisdiction-wide smoke-free workplace policy by NT Department of Health: “ *The new structure and operations systems will make it easier to implement and enforce workplace and environmental policies”* (FLSPNI).

The current Territory and Commonwealth Governments’ social and economic development policies [13,20], i.e. other than those targeting tobacco, were mentioned as a *challenge* and are discussed more fully under the heading ‘Group 3: stress’.

**‘Health workforce capacity’:** Although identified frequently as an *opportunity* (n = 15) this was also highest on the *challenge* continuum (n = 24) (Figure 2). Providing further capacity-building resources and opportunities for the community-based health workforces was seen as a major *opportunity* for contributing to addressing tobacco related issues. In particular mention was made of capacity building among those staff who were local Aboriginal community members: “ *Local staff are the constant. They should have ownership”* (FLSPNI). A service provider expressed the concern: “ *There are less Health Workers in the NT than other jurisdictions – they are disappearing like flies”* (FLSPNI). NT Health Department policies at the time of interviews were noted as an *opportunity* to increase capacity of Indigenous employees through *“good policies to try and encourage growth in the Indigenous component of the workforce with a flow-on effect, for example the cross-fertilization of ideas and knowledge”* (NFLSPNI). There were indications that this can be difficult to realize at the local level. ” *There’s a lack of support or mentoring for* [Indigenous] *Health Workers when acquiring new skills*” (FLSPNI).

Further issues cited as *challenges* were the heavy workloads for nurses in community health centres with multiple programs and difficulties retaining clinical staff in remote communities. Non-indigenous staff noted that staffs with specialist drug and alcohol skills were often irregular visitors to the communities: “ *There is a lack of tobacco-dedicated staff – need a local person with local knowledge”* (FLSPNI).

#### Group two: Often seen as an opportunity, not often as a challenge

This group contains themes grouped at approximately the same frequency on the *opportunity* axis but not often mentioned as *challenge* (Figure 2). This group includes the themes: ‘workplace interventions’ and ‘role models’. “Research’, ‘school-based interventions’ and ‘community engagement’ were mentioned with equal frequency as an *opportunity* (n = 9) (see Figure 2).

**‘Research’:** Tobacco research, community-based research in particular, was identified as an opportunity by both Indigenous and non-Indigenous participants, particularly FLSP’s. It was not mentioned as a *challenge.* ‘Research’ was described as having potential to *“…provide data to funding bodies*” (NFLSPI); “ *…evaluate the effectiveness of local programs”* (FLSPI) and to *“…raise community awareness about tobacco-related harms”* (FLSPNI).

**‘Community engagement’:** Recommendations made to maximize *opportunity* included health staff increasing efforts to work outside of the clinic to improve engagement with community members and make better linkages with local community organisations. One clinic worker noted he had “ *opportunities to go out bush and to outstations and talk with people in a more relaxed situation than in the main community”* (FLSPI) *.* Cited as an example of community engagement by a regionally-based drug and alcohol service was their “ *…outreach programs to remote communities and to colleges with boarders from remote communities* [i.e. children from remote communities in boarding schools]” (FLSPI).

**‘School-based interventions’:** Mentioned as an *opportunity* only by FLSP’s these mainly focused on the provision of tobacco education by both teachers and visiting service providers: “ *Send a messenger to the school monthly doing smoking education. Let them know smoking is the number one killer – that most men die in their forties”* (FLSPI). There was also mention of smoke-free policies for school campuses. *Challenges* to these interventions included: “ *Tobacco is not the core business of the school”* (FLSPNI). *There has been a lot of noise … about tobacco on school grounds. Little has actually happened to address tobacco issues. It’s probably been in the too hard basket*” (FLSPNI).

**‘Workplace interventions’:** Strategies identified as “ *providing a supportive environment for staff to quit*” (FLSPNI) included the provision of quit support, setting up designated smoking areas or making workplaces totally smoke-free. At the time of interviews the workplaces caring for infants and elderly in the study communities were most effectively implementing smoke-free policies. A local government manager supervising staff in Council functions thought there was potential to “… *include tobacco education for staff as part of the induction process*” (FLSPNI). The only potential *challenge* to tobacco interventions in these settings mentioned was the service-related cost for the provision of quit support **.**

**‘Role models’:** Positive local role models were identified as a potentially effective strategy (Figure 2): “*Strengthen the blokes* [men] *that can help. Non-smokers can come and sit. They have more power to help. Ex-smokers can tell their experiences: how they stopped*“ (FLSPI). However, poor role modeling by some parents, local Indigenous Health Workers, nurses, teachers and other non-Indigenous service providers was conversely noted as a challenge: “ *Parents are poor role models smoking. Little grandkid picks up sticks and pretends to smoke”* (FLSPI). Aboriginal Medical Services were recommended “ *to make quit support services available for staff. It’s not good to be doing brief interventions in tobacco* [in a health service] *and be smelling of cigarette smoke”* (FLSPI).

#### Group three: Not often seen as an opportunity but often seen as a challenge

Themes in this group all clustered around a low frequency of mention as an opportunity (Figure 2).

**‘Supply reduction’** was mentioned as an *opportunity* only by Indigenous participants: “ *Best way to stop with smoking is to stop where the smoke is coming from – the tobacco companies*” (FLSPI). “ *Stop selling tobacco at the store….by taking it away, within two generations the kids won’t have been exposed*” (FLSPI). “… *restricting availability of tobacco at stores to 2–3 days per week only*” (FLSPNI). Responsibility for driving these strategies was seen to be local, particularly the committees responsible for the governance of local community stores.

**‘Collaborative efforts’** between local agencies were mentioned most often as a *challenge*. “ *All service providers* [in the community] *have their own focus*” (FLSPI). “ *People are working in silos and not allied teams*” (FLSPI).

**‘Health services’** were seen to be *challenged* because of insufficient funding for appropriate levels of staffing and program resources locally. Frequent use of the ‘ *fly-in, fly-out*’ service model was seen as problematic with visiting staff covering huge geographical areas exacerbated by a lack of suitable accommodation facilities restricting time spent in the communities. The resultant brief visits were regarded as limiting for community engagement. In remote communities, access to the health workforce is largely through the local clinic where the priority for service is usually “ *dealing with acute issues – tobacco isn’t a priority*” (FLSPI) *. ”The clinic is not the place that people come asking for quit assistance”* (FLSPNI). Government health services were seen as an opportunity for advocacy with a major role *“in empowering levels of responsibility and control to enable people to make their own choices. Community members are sick of being told what to do”* (FLSPNI).

**‘Cultural issues’** were mentioned as *challenges* mostly by those working at the community level. In one of the communities there are strong cultural influences relating to knowledge and use of tobacco arising from a history of trade with Macassan fishermen from Indonesia prior to colonization [21]. During early community consultations, a prominent community elder informed the researchers that: “*Tobacco use is embedded in our culture, you won’t be able to change it”.* In the dual moiety system of Arnhem Land, the Yirritja moiety members have particular responsibility for keeping tobacco and smoking knowledge and ceremonies. Some perceive that quitting tobacco use interferes with adhering to these cultural responsibilities. On the other hand, those of the Dhuwa moiety declare: “ *I can’t leave this* ( *smoking) because ngarali* ( *tobacco) is my waku* (child).’” (FLSPI). Yirritja ownership of tobacco was not necessarily seen as a barrier to people quitting smoking. “ *The Yirritja ngarali story is about the feeling and about the crying, not about smoking”* (FLSPI). The sole *opportunity* mentioned in the context of this theme is that these peculiar cultural arrangements provide the prospect of a return to using old cultural laws to limit the use of tobacco in some subgroups in the local community and to limit daily consumption.

The remaining themes were mentioned only in relation to *challenge*.

**‘Use of tobacco with other drugs’** commented on only by FLSP’s, the majority non-indigenous. “ *Gunja* [cannabis] *is mixed with tobacco – you have to consider them together. When there’s no gunja, people pack bongs* [smoking utensils] *with just tobacco”* (FLSPNI). Participants advised this should be acknowledged and addressed in both education and quit support programs **.** Frequently commented on was: *“people are more likely to smoke when they are drinking* [alcohol] *”* (FLSPI) *.* There was also mention that it is: “ *hard not to smoke around kava* [a traditional drink from the Pacific used in one of the study communities since the 1980s [22]. *You smoke like a train then*” (FLSPI).

**‘Geographical issues’** were most frequently mentioned as a *challenge* by participants not living in the communities. “ *In practice it is hard to deliver any programs in the communities – we have a huge area to cover with two staff – the logistics are impossible*” (FLSPNI). Regionally-based staff frequently travel huge distances, limiting time spent in the communities: “ *they cram their work in*” (FLSPI). Isolation and limited amenities for community-based staff were identified as contributing to frequent staff turn-over (NFSPNI).

**‘Stress’** was frequently mentioned by those working and living in the communities as a *challenge* to reducing tobacco consumption. Socio-economic contributors to this stress include high levels of unemployment, inadequate housing, limited services, high food costs and the impacts of colonization during the early part of the 20^th^ century in Arnhem Land. “*There is a high prevalence of mental health issues –people are self-medicating with tobacco”* (FLSPNI). Some stresses mentioned peculiar to these communities mainly relate to the recent major policy changes around local governance, and Commonwealth Government interventions precipitated by a report on the safety of children [23]. The impact of these reforms was thought to be leading to increased tobacco consumption especially by decision-makers: “*Stress is so constant. Matters between clan groups and shire change. People carry a lot on their shoulders. Community leaders are smoking more due to these stresses*”(FLSPI).

**‘Tobacco is not regarded by many people at the community level as a priority’**: **“***Tobacco is not an in your face problem like alcohol, petrol sniffing”* (FLSPI), “ *Cannabis is seen as the greater problem and children engaging in sniffing and truancy from school… these distract from the smoking issues“* (FLSPNI). “ *Clinics are dealing with acute health issues – tobacco is not a priority”* (FLSPI).

**‘Prevalence of tobacco use’: “***Indigenous people working in the communities…especially where there is greater than 80% smoking rate, feel there is no way out. No way of changing the smoking epidemic that is helping to suck the livelihood from the most marginalized*” (FLSPI). The high prevalence rate is regarded as underpinning normalization of tobacco use and contributing to the strong triggers or cues to continue smoking.

## Discussion

These results provide a unique picture of the entrenched inequalities between these isolated Indigenous communities at the extreme margins of a high-income country, Australia. These disparities make for comprehensive challenges to effectively implementing tobacco policy which inevitably is developed far away in Australia’s capital cities. The main *challenges* are: very high rates of smoking which appear to be normalized, cultural and language barriers to delivering relevant health promotion information, health services and staff under-resourced to provide the most basic tobacco interventions, schools ill-equipped to prevent the uptake of tobacco, high levels of stress with reduced local control over local community governance and management and the extreme remoteness of these isolated populations from services normally available to other Australians in the urban centres. Policies targeting smoking in these remote Indigenous settings need to be more effectively implemented as a matter of urgency.

National policy documents targeting a reduction of tobacco-related harms among Australia’s Indigenous peoples [9,24-26] have major strategies in common. These include commitments to workforce development; social marketing campaigns; improved delivery of cessation support and stronger regulatory efforts with a focus on reducing exposure to second-hand smoke. As part of ‘Tackling Smoking’ there is a national network being developed of appropriately trained tobacco action workers to support Indigenous communities, although few of these are currently based in remote communities [27]. There is an upsurge in national, regional and local social marketing campaigns targeting Indigenous peoples to challenge the acceptance of smoking as normal behaviour.

Considering the results of interviews with the 82 participants in this study, it is clear that this current national policy framework is congruent with what frontline service providers are saying is needed in the communities. However it is equally clear from these results that policy implementation at the remote community level remains a *challenge.* It is therefore important to consider the best way to invest in the *opportunities*, and the best approach to overcoming the *challenges*.

As our data have indicated, strategies relating to smoke-free policies, smoking cessation support and health promotion were themes most frequently mentioned as *opportunities*. Evidence from evaluations of the effectiveness of smoke-free policies in settings other than remote communities suggest that cigarette consumption in adults and youth and exposure to second-hand smoke can be reduced and can have positive impacts on cardiovascular disease [28]. There are indications in the results that robust smoke-free policies would be welcome in the study communities. Comments such as “*Smoke free policies in clinics: the policy has mostly worked well at the regional hospital and has helped some staff to either quit or cut down”* (FLSPI) reflect both community members’ and service providers’ views that enforced smoke-free policies, particularly in workplaces, may be effective for reducing consumption *.*

Smoking cessation and support services tailored to meet the needs of Indigenous peoples, particularly those living in remote communities where prevalence rates are the highest in the country, are slow to develop. Currently the few remote community-based tobacco workers have limited capacity to provide quit support. Early consultation in the TETP with communities demonstrated a preference for face-to-face support, delivered by community members who spoke their language. Daily tobacco consumption information was obtained from 177 smokers in TETP baseline tobacco surveys in the study communities. Of these, 55% reported smoking 10 or more sticks per day, indicating they may benefit from more intensive quit support recommended by current clinical guidelines i.e. counseling, pharmacotherapy or a combination of these [29]. Government-subsidised cessation medicines, supplied in local community health centres, include only trans-dermal NRT and come with the requirement that recipients of the subsidised therapy have*“…entered into a comprehensive support and counseling program*” [30] Clearly further pharmacotherapies, such as nicotine gums should be more readily available on a subsidized basis in these impoverished communities. At the time of the baseline surveys in 2008/2009 some participants thinking about quitting stated that they didn’t know where to go for help. Despite more recent policy commitments at Commonwealth and Territory levels, appropriate and accessible quit support for smokers in these settings remains limited.

Centralised telephone counseling services provide a cost-effective service with a wide geographical reach [31] well-suited to the vast areas of service delivery in remote Australia. Such services are more effective if integrated with concurrent population-based approaches to reduce tobacco use. In several Australian jurisdictions efforts are being made to tailor such services. A model currently being developed in Western Australia (WA), initially for Aboriginal people living in a metropolitan area, contains components that could be used in remote communities. These include comprehensive community engagement and service promotion by senior Aboriginal staff, and incorporation of local knowledge into providing support or referrals [personal communications JR 2012: J Keene & P Parfitt, Drug & Alcohol Office, WA]. While such a service cannot provide face-to-face counseling with individuals in remote communities, it could provide face-to-face engagement with the community through occasional site visits to promote the service.

Opportunities mentioned for health promotion activities in these settings were mainly related to addressing the lack of understanding of the impacts of tobacco use. There is a need to consider historical and cultural origins of tobacco use, accurate word meanings and Indigenous concepts in order to effectively build and exchange tobacco knowledge [32,33]. In the experience of our team, this means taking a dialogical rather than a didactic approach [34].

Indigenous Health Workers are a vital component of the health workforce particularly in remote communities. Usually members of the communities they are working in, they are able to communicate in local languages, they provide translation of knowledge, community knowledge and linkages as well as a range of clinical services. While there are concerted efforts to up-skill a range of health professionals through workshops, generally held in regional rather than remote settings, access to information provided remains limited for those health workers from remote communities. The non-Indigenous health workforce in these settings requires support to enable an increased focus on addressing chronic disease risk factors such as tobacco. This would entail further investment in mentoring relationships incorporating reciprocal learning between Indigenous and non- Indigenous staff to tackle tobacco [35].

### Limitations of the study

In this study we looked for ideas and statements believed by participants to be *opportunities*. *Opportunities* are not the same as what participants might have identified as the most effective strategies. Policy implementation could address this specifically as programs are developed. A second limitation of the study is that participant recruitment may have been influenced by researchers’ bias if, unintentionally, study participants were selected because of existing professional relationships and networks. However, in order to minimize this, we took advice from participants asking them to direct us to others for interview following their recommendations diligently. As in several previous studies in this region, conducting interviews in plain English without audio-recordings was not a limitation [4,5].

The number of participants interviewed was small and over a short time frame. The results of the study therefore should be viewed as a ‘snapshot’ pertaining to just one time point when tobacco policy and program implementation targeting Indigenous smoking had unprecedented momentum. Standing against this limitation is the unique picture we have provided of community level views about implementing tobacco policies during perhaps Australia’s most dynamic period of tobacco policy generation to address high and unchanging rates of smoking in these disadvantaged populations. The picture provided by our data is comprehensive because we believe we achieved saturation across a large part of the key individuals and agencies working across the NT with tobacco as their core business. Importantly, even though the sample was small we had representation of views from most key policy implementers, especially those at the front line in these three remote communities.

The responses of frontline service providers, both Indigenous and non-Indigenous, clearly demonstrated their deep understanding of local community issues and their thoughtfulness about the *opportunities* and *challenges* faced at this practical level of service delivery. Those interviewed who were not working at the frontline but at senior executive or management levels had potentially more impact on policy implementation decisions yet had less knowledge about community level issues.

### Recommendations

On the strength of the high level of need and based on the reported results and discussion, it is recommended that in these remote settings:

· whole of community approaches are used to improve collaborative efforts between service providers, extending beyond those with health as core business

· cessation support is enhanced by 1) access to a locally-based Indigenous tobacco-specific workforce equipped to confidently provide quit support and information about tobacco-related harms utilizing local language and conceptual frameworks; 2) increased access to a wider range of subsidized pharmacotherapies to assist smoking cessation, including short-acting nicotine replacement products; 3) centralized phone services are funded to provide face-to-face service promotion of their activities in remote communities as an engagement strategy

· community-based support is sought for regulatory efforts to focus on the reduction of exposure to second-hand smoke and

· workplaces are a focus for interventions.

There is some congruence between the above cessation support recommendations and those based on findings from studies undertaken among Indigenous peoples in New Zealand and Canada [36-38]. The above recommendations may also find a place in low- and middle-income countries struggling with rising cigarette sales in an environment of social and cultural diversity [39]. Strategies more specific to the remote Indigenous populations in the NT include recommendations that further place-based approaches including schools are considered and that concomitant cannabis smoking is acknowledged and addressed in tobacco interventions. Further research is needed across a range of disadvantaged subpopulation groups to rigorously evaluate specific components of community-based interventions.

## Abbreviations

LIP, Local Implementation Plans; NPA, National Partnership Agreement; NPARSD, National Partnership Agreement on Remote Service Delivery: NT, Northern Territory; TETP, Top End Tobacco Project; FLSPI, Frontline Service Provider Indigenous; FLSPNI, Frontline Service provider Non-indigenous; NFLSPI, Non-Frontline Service Provider Indigenous; NFLSPNI, Non-Frontline Service provider Non-Indigenous; WHO, World Health Organisation..

## Competing interests

The authors declare they have no competing interests.

## Authors’ contributions

The study was conceived and designed by JR, undertaken by JR and AC, JR lead the analysis guided by AC; JR lead the writing guided by AC, KC and RI. All authors read and approved the final manuscript.
